# The two faces of microorganisms in traditional brewing and the implications for no- and low-alcohol beers

**DOI:** 10.3389/fmicb.2024.1346724

**Published:** 2024-02-19

**Authors:** Giulia E. Roselli, Daniel W. M. Kerruish, Matthew Crow, Katherine A. Smart, Chris D. Powell

**Affiliations:** ^1^Division of Microbiology, Biotechnology and Brewing Science, School of Biosciences, University of Nottingham, Loughborough, Leicestershire, United Kingdom; ^2^Diageo Ireland, Dublin, Ireland; ^3^Diageo International Technical Centre, Menstrie, Scotland, United Kingdom

**Keywords:** yeast, bacteria, contamination, microbiological spoilage, beer quality

## Abstract

The production of alcoholic beverages is intrinsically linked to microbial activity. This is because microbes such as yeast are associated with the production of ethanol and key sensorial compounds that produce desirable qualities in fermented products. However, the brewing industry and other related sectors face a step-change in practice, primarily due to the growth in sales of no- and low-alcohol (NoLo) alternatives to traditional alcoholic products. Here we review the involvement of microbes across the brewing process, including both their positive contributions and their negative (spoilage) effects. We also discuss the opportunities for exploiting microbes for NoLo beer production, as well as the spoilage risks associated with these products. For the latter, we highlight differences in composition and process conditions between traditional and NoLo beers and discuss how these may impact the microbial ecosystem of each product stream in relation to microbiological stability and final beer quality.

## Introduction

Since its conception, beer has historically been one of the most broadly consumed beverages worldwide. Aided by technological innovations, brewing has developed from a simple process, involving the contribution of a range of naturally occurring microbes, into a multistage industry where desirable and specific microbial activity is dictated and closely controlled. Indeed, product type and brand specifications are largely determined by the activity of proprietary yeast strains in pure form (i.e., monocultures) during the fermentation step. While yeast activity is closely associated with alcoholic beverage production, other microbes can be applied to manipulate the properties of raw materials and substrates (wort) prior to fermentation, while some can be used to lend certain characteristics to beer post-fermentation. However, at most stages of the process, the presence of microorganisms other than the production yeast strain is undesirable and efforts are taken to eliminate, prevent, or supress their growth. Unfortunately, contamination of brewery raw materials, beer in process, and final products can occur throughout the supply chain, from the field through the production plant and to the point of consumption. This is of major significance to industry as it has been estimated that the European alcoholic and non-alcoholic beverage sector alone suffers an economic loss in the range of millions to billions of Euros per year due to microbial spoilage (Stratford, 2006).

In recent years the alcohol-free drinks market has seen a rapid expansion, primarily driven by demand from consumers seeking to lead a healthier lifestyle by moderating alcohol consumption. The alcohol-free beer market has been at the front of this transition since product diversification also offers the opportunity to expand the consumer base to include non-traditional drinkers as well as those affected by temporary (pregnancy, breastfeeding, driving, operating machinery, sports involvement) or more permanent pathological conditions (liver disease, alcoholism, cancer, medication), incompatible with alcohol consumption. Furthermore, alcohol-free products provide the potential for breweries to enter markets in countries where alcohol consumption is illegal or discouraged for religious reasons. Sales of beverages in the no- or low-alcohol products are increasing and currently represent the fastest-growing sector of the global beer market, expected to grow annually and be worth $40 billion (USD) by 2032 (Pulidindi and Ahuja, 2022). It should be noted that the precise definition of a no- or low-alcohol (NoLo) beer is not universal, and the defining values with respect to alcohol content vary between Europe, United States and elsewhere (Quain, 2021). For the purpose of this article the terminology used will refer to UK legislation; no-alcohol (No) beers are labelled as containing ≤0.05% ABV and low-alcohol (Lo) beers ≤1.2% ABV (GOV.UK, 2018).

Due to growth in the NoLo brewing sector, global brewing companies and small-scale brewers are increasingly required to produce multiple product types using essentially the same raw materials, apparatus, processes, and packaging facilities. During standard beer production, the action of yeast during the fermentation step functions to create a microbiologically stable product with a series of antimicrobial ‘hurdles’, including hop bitter acids, low pH, lack of nutrients, low oxygen, elevated concentrations of carbon dioxide, and the presence of ethanol. The elimination of ethanol from this list (in the case of NoLo products) alters the niche significantly, such that microorganisms not typically associated with beer may be able to survive and proliferate. Since NoLo beers are therefore at greater microbiological risk than traditional beer types, brewers face a challenge to ensure that products released to market are of high quality and stable over time.

In this review, we provide an overview of the contribution of microbes to the brewing process. In the first section we consider the different ways in which microorganisms can contribute positively towards beer production. It should be noted that although the contribution of production yeast to fermentation is mentioned briefly here for context, more comprehensive information can be found elsewhere. In the second section we discuss the negative implications of unwanted microbes across the brewery and the environmental conditions that affect their growth, survival, and spoilage potential. In the final section, the key characteristics of NoLo products are described, and their spoilage potential is discussed in light of current microbiota, as well as with regards to other organisms not presently associated with beer and brewing. Although focused primarily on the brewing sector, this information can also be applied to related industries experiencing similar trends and with similar production demands.

## The positive contribution of microbes to brewing

Currently, a variety of yeasts and bacteria are used in the production of a broad range of beer styles. In the majority of instances, *Saccharomyces* yeasts are responsible for primary fermentation. The individual strain employed in alcoholic fermentation has a direct and specific impact on the nature and characteristics of the final product. However, secondary microbes can be utilised throughout the brewing process for a variety of reasons: to enhance the processability of raw materials, to provide a unique character to substrates, to improve or contribute to production efficiency, or to modify the final product post-fermentation. Such contributions are well established in other related industries, for example the use of malolactic bacteria in wine production (Lasik, 2013), and the symbiotic relationship between *Aspergillus* (koji) and yeast during sake fermentations (Ito et al., 2023). However, within the brewing sector alternative uses of microorganisms is limited to niche product types and novel practices, many of which are still in development.

### Microorganisms in malting

Barley grains, used for the production of malt, host a diverse microbial population. This microbial ecosystem can be influenced by a variety of environmental factors (including climate, plant variety, cultivation, storage conditions), however it usually consists of bacteria, yeasts, and filamentous fungi (Noots et al., 1999). While these microorganisms and their metabolites pose a potential risk throughout the brewing process itself, the positive influence of this microbial ecosystem on malt characteristics is noteworthy. The metabolic regulation of barley germination is influenced via plant growth regulators, and various microorganisms are involved in the production of metabolites including plant hormones that promote germination (Tuomi et al., 1995; Flannigan, 1996a; Noots et al., 1999). In addition, microbes can provide vital sources of amylolytic, proteolytic, and cell wall-degrading enzymes (Noots et al., 1999; Hammes et al., 2005). Moreover, microorganisms increase the nutritional content of malted barley by breaking down anti-nutritive components and enhancing the bioavailability of key compounds including vitamins, minerals and proteins (Hammes et al., 2005). Certain strains of malt-derived lactic acid bacteria (LAB), typically considered to be beer-spoiling organisms, have been shown to positively influence the quality and safety of malt, and malt-derived products (Laitila et al., 2006). Inoculating and/or processing the barley with a starter culture of microorganisms that have specific characteristics such as antimicrobial properties remains a developing technology (Haikara and Mattila-Sandholm, 1997; Iserentant et al., 2010; Malanda and Boivin, 2012), however this practice is both technically and economically feasible. The inhibitory activity of LAB occurs due to increased competition for nutrients and space, and through synthesis of antimicrobial compounds including organic acids (i.e., lactic and/or acetic acid) hydrogen peroxide, bacteriocins, and other low molecular weight antimicrobial compounds (Ouwehand and Vesterlund, 2004). Alongside these antimicrobial properties, the use of LAB can be implemented in the production of biologically acidified malt, mash, or wort (Laitila et al., 2006), and similarly to alter the technological and organoleptic properties of malt, wort and the final beer (Pittner and Back, 1995; Lowe et al., 2004, 2005). There are a variety of advantages to carrying out mash and wort acidification, for example, where low quality malt is used, mash acidification can offset the lower enzyme activity typically associated with this grade of material (Lowe et al., 2004). The reduction in pH promotes malt enzyme activity (i.e., limit dextrinase), and at the same time, the activity of β-glucan solubilases is inhibited, enabling a lower mash viscosity and as such producing a mash with greater lautering performance. Biological synthesis of lactic acid by bacteria can be challenging to optimise and control, and alternative approaches have been investigated, including the use of *Lachancea theromtolerans* as a yeast alternative to LAB for the production of lactic acid for brewing purposes (Domizio et al., 2016). Data has also indicated that *L. theromtolerans* can be utilised alongside *Saccharomyces* in brewing fermentations to produce a rapid decrease in pH (Gobbi et al., 2013), while those strains that produce greater quantities of lactic acid may find direct application in sour beer production (see below).

### Microorganisms in brewing fermentations

**Figure 1:**
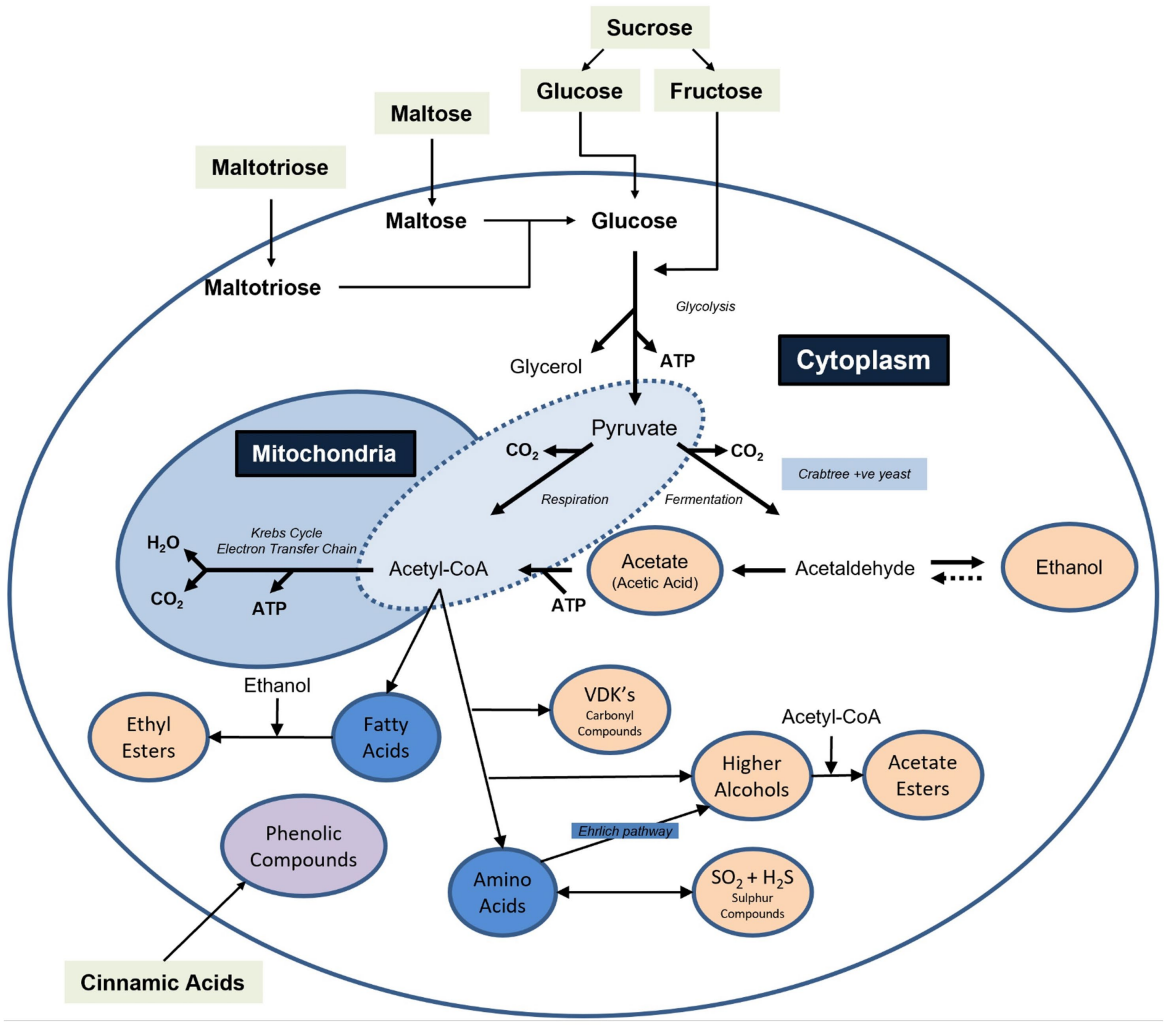
A simplified overview of the key metabolic products generated by yeast strains during fermentation. Note that the extent and ratio of compounds are often interlinked and dependent on factors such as the nutritional content of the wort, the nutritional requirements of the yeast strain, as well as factors influencing yeast growth such as inoculation rate and the amount of oxygen available. Note also that in some yeast species certain pathways may be used preferentially, while others may be absent. For example, for *Brettanomyces* yeast strains, the ability to produce glycerol is limited and the production of phenolic compounds is greatly elevated when compared to *Saccharomyces* yeasts. Created with Biorender.com.

The key sensory attributes of beer are developed during the fermentation process, as a result of microbial activity. This activity can serve to reduce or increase the concentrations of wort compounds, or to convert nutrients into entirely new products. Key examples include the production of ethanol, as well as flavour-active compounds including higher alcohols, esters, carbonyl compounds (especially vicinal diketones), sulphur compounds and phenolics. To achieve these changes, a range of organisms can be used in controlled brewery fermentations, however *Saccharomyces* yeast strains are most commonly employed. The preferential use of these ‘conventional’ yeasts is due to their efficiency, robustness, versatility, and capacity to produce a balanced range of flavour compounds as intermediates or by-products of metabolism. Yeast strains belonging to the species *Saccharomyces cerevisiae* are typically used for the production of ale type beer, while lager products are, by definition, produced as a result of fermentation using the hybrid organism *Saccharomyces pastorianus*. The role of *Saccharomyces* yeast in fermentation is extensively documented in the literature and will not be covered in detail here, however the main metabolic products formed by yeast can be found summarised in Figure 1.

Previously a niche activity, in recent years the use of ‘non-domesticated’ or ‘wild’ *Saccharomyces* and non-*Saccharomyces* yeasts for the production of beer has gained interest among the brewing and scientific community, largely in response to demand for flavour and product diversification. Application of non-*Saccharomyces* yeasts in controlled fermentation processes can impart novel flavour characteristics to the final product, control microbial spoilage, and/or modify other important parameters such as final alcohol content (Steensels and Verstrepen, 2014). As for standard yeast types used in beer production, a range of flavour-active compounds can be produced, and their creation is typically species and strain specific (Pires et al., 2014); the use of wild yeasts provides a natural mechanism by which flavour profiles can be enhanced or altered, giving rise to speciality beers that have unusual characteristics. Examples include the production of sour beers, lambic beer, gueuze, and those with ‘funky’ character; a term which broadly describes a variety of aroma compounds such as ‘band-aid’, ‘mousy’, ‘barnyard’ or ‘horse blanket’ arising primarily from the presence of phenolic compounds (Steensels et al., 2015).

The use of ‘wild’ organisms in brewing reflects an evolution of natural fermentation techniques. Certain beer styles have historically been produced using *‘*starter’ cultures that can be considered to be either non-spontaneous, where wort is inoculated with an autochthonous starter culture, or spontaneous, where microbes are introduced into the system via air-inoculation of wort, often using a shallow open vessel termed a ‘coolship’. This process allows a mixture of brewery-resident yeasts and bacteria to naturally inoculate cooled wort that is subsequently run into large oak barrels (foeders) and allowed to ferment. Over time the microbial population dynamics shift, often depending on product type, raw materials, fermentation equipment and geographical location. However, it has been shown that individual breweries develop their own microbiota and that the resident brewhouse microbiota is largely responsible for conducting the fermentation (Bokulich et al., 2012). Previous studies have indicated that over the first month of spontaneous fermentation the microbial ecosystem predominately consists of non-*Saccharomyces* yeasts, and some *Enterobacteriaceae,* including *Klebsiella*, *Enterobacter, Escherichia, Citrobacter, Serratia,* and *Pectobacterium* (Martens et al., 1991; Bokulich et al., 2012). Subsequently, a decrease in pH results in a reduction in overall diversity and a selection for lactic acid bacteria (LAB), predominately *Pediococcus*, while reduced competition for nutrients gives rise to an increase in the number of *Saccharomyces* yeasts. Correspondingly, the main fermentation is undertaken by the LAB and the *Saccharomyces* yeast, usually for a period of 3 to 4 months. After this point other types of yeast such as *Brettanomyces* begin to dominate as these are able to gradually super-attenuate the beer during the long maturation phase (Bokulich et al., 2012). The *types* of flavour compounds produced are broadly in line with those depicted in Figure 1, however it should be noted that the ratio and *extent* to which they are produced differ when compared to production by ‘domesticated’ yeast strains (Steensels et al., 2015).

### Applications of yeast cultures post-fermentation

Some brewers may choose to conduct a ‘secondary’ fermentation at cold temperatures, typically directly within a cask, or bottles used for packaging. This process is usually associated with standard ale type beers and is sometimes referred to as ‘refermentation’(Van Zandycke et al., 2011). The aim of secondary conditioning is to generate carbon dioxide and ethanol, while also allowing the removal (by yeast) of any oxygen that may be present. At the basic level, refermentation can be achieved by transferring beer and yeast immediately prior to the end of fermentation; at a stage where some residual fermentable sugars remain. Alternatively, fully fermented beer can be seeded with a bespoke yeast culture (typically a *Saccharomyces* yeast) along with some additional ‘priming’ sugar with similar results. Irrespective of the approach, this process functions to ‘stabilise’ the beer by removing oxygen to prevent staling over time, while also allowing some desirable flavour changes to occur, as determined by the yeast strain employed (Stulikov et al., 2020).

## The negative aspects of microbes to brewing

### Microbial contamination within the brewing chain

Beer is resistant to the growth and survival of microorganisms and has historically been considered to be a product that is safe for consumption, since it is not associated with organisms that cause foodborne illnesses. This is due to a combination of a series of intrinsic antimicrobial characteristics including the presence of ethanol, hop bitter compounds, low pH, high CO_2_ concentrations, low O_2_ content and a general lack of nutrients such as fermentable carbohydrates, vitamins and amino acids (Sakamoto and Konings, 2003; Menz et al., 2009). Extrinsic factors relating to the brewing process itself also lower the risk of contamination or proliferation by microorganisms. These include the acidification of malt, the mashing process, wort boiling, pasteurisation, filtration, and cold storage (Vriesekoop et al., 2012). The combination of intrinsic and extrinsic factors described provide a prime example of hurdle technology (Leistner, 1985), where a combination of obstacles that may not be individually restrictive, are together able to prevent contamination. This results in a product with enhanced shelf-life and stability, while key desirable organoleptic properties are not compromised. The main targets and the mode of inhibition of key antimicrobial hurdles present in beer can be found in Table 1. It is important to note that the same principles apply when considering the survival and growth of beer spoilage microorganisms, as well as potential pathogens as discussed further below.

**Table 1:** Targets and inhibition mechanisms of both intrinsic and extrinsic antimicrobial hurdles of beer.

Antimicrobial hurdles		Limits	Mode of inhibition^b^
*Intrinsic*	Ethanol	0.5–10% (v/v)^a^ On average 3.5–5.0% (v/v)^b^	Reduces cell membrane capability
	Low pH	3.8–4.7^a^	Impacts enzyme action, Improves inhibitory action of hops
	Hop bitter compounds	~ 17–55 ppm iso-α-acids^a^	Reduces cell membrane capability, Only inhibit Gram-positive bacteria
	High CO_2_ concentration	~ 0.5% w/w^a^	Produces an anaerobic environment, Lowers pH, Impacts enzyme action, Impacts cell membrane
	Low O_2_ concentration	< 0.1 ppm^a^	Produces an anaerobic environment
	Absence of nutritional substrates	Traces of glucose, maltose and maltotriose^a^	Starves cells
	Sulphur dioxide	SO_2_ originates predominately from yeast metabolism Maximum permitted level of total SO_2_ is 20 mg/L^c^	Impacts several metabolic systems
*Extrinsic*	Mashing		Triggers thermal destruction of cells
	Wort boiling		
	Pasteurisation		
	Filtration		Eliminates cells by physical size exclusion
	Bottle conditioning		Produces an anaerobic environment

a Information summarised from Sakamoto and Konings (2003).

b Information summarised from Vriesekoop et al. (2012).

c Information summarised from Dvořák et al. (2006).

**Figure 2:**
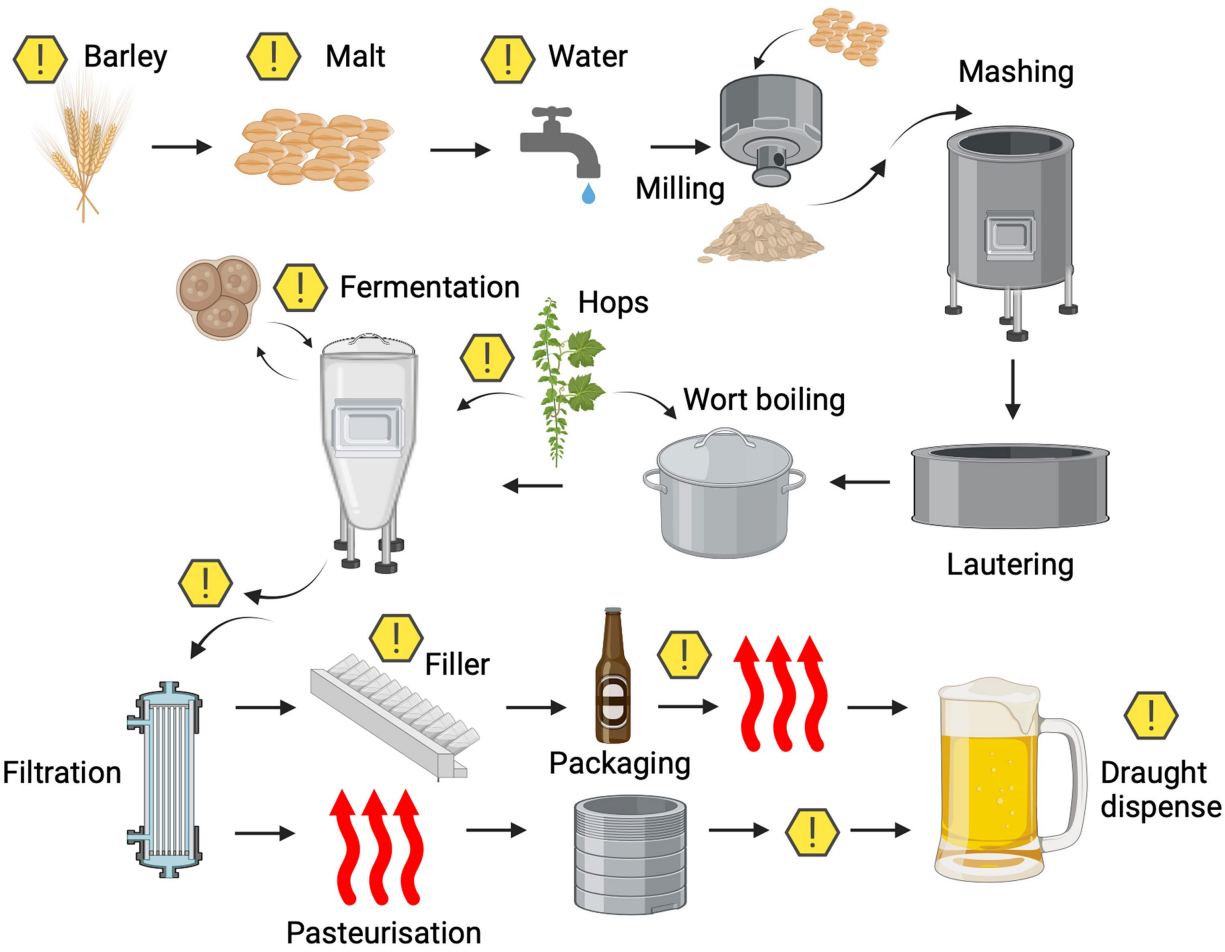
Microbiological points of risk typically observed in the brewing process. Potential sources of microbial contamination are indicated by the warning symbols. Created with Biorender.com.

**Figure 3:**
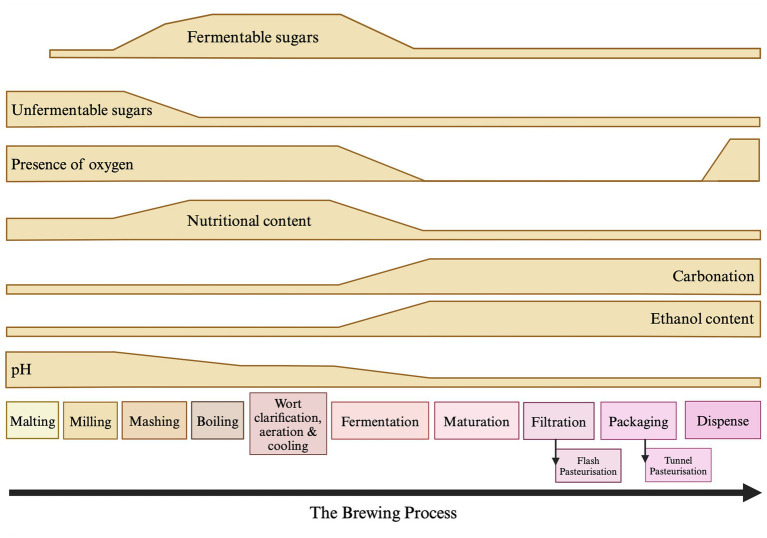
Environmental and physiochemical factors associated with the brewing process that may influence the risk of microbial spoilage. The changes that occur in un/fermentable sugars, oxygen, nutritional content [including free amino nitrogen (FAN) vitamins and minerals], carbonation, ethanol content and pH are represented. Boiling and pasteurisation (when used) represent key stages where microbial loading is reduced. Note that additional factors such as microbial adaptation to stress should be considered, while effective sanitation and correct storage temperatures are relevant across the brewing process. These latter should be controlled where possible, otherwise risk can be exacerbated accordingly. Created with Biorender.com.

Microbial contaminants that are able to spoil beer can enter the process at various stages and can be derived from many different sources (Figure 2). These microorganisms can be broadly split into two categories and defined as either primary or secondary contaminants. Primary contaminants are those originating from the raw materials (hops, cereals, water, and priming sugars) and brewhouse containers, while secondary contaminants are those established during bottling, canning or kegging (Vaughan et al., 2005). Approximately half of the reported cases of microbial contamination are believed to occur due to secondary contaminants, typically as a result of inadequate cleaning and sanitation methods. However, primary contaminants are often considered to be more dangerous, as they can jeopardise entire production runs (Vaughan et al., 2005) and can present recurring issues if not eliminated. A summary of the typical beer spoilage organisms and the stage of the brewing process at which they are associated can be found in Table 2. It should be noted that while it is convenient to associate ‘risk’ with process stages, it is also important to consider the intrinsic and extrinsic physiochemical factors that influence microbial growth. A broad indication of how these environmental parameters change over the course of the brewing process can be found illustrated in Figure 3. It should be noted that these can inevitably vary, depending on individual breweries, product streams, and the processing strategies for raw materials and end products. However, ultimately these combined factors provide information that can form the basis of sampling and detection plans, since the environmental conditions at each stage provide a general indication of which organisms may/may not be present.

**Table 2:** Common beer spoilage species and their effects on beer.

Stage of brewing process	Spoilage organism^b^	Information	Literature
Brewing raw materials	*Aspergillus fumigatus*	Off-flavours: roughness and stale	Vaughan et al. (2005)
	*Fusarium culmorum*	Production of mycotoxins, gushing inducer	Laitila et al. (2002)
	*Fusarium graminearum*		
Mashing & wort separation	*Ped. pentosaceus*	No defect in finished beer detected	Simpson and Taguchi (1995)
	*Bacillus coagulans*	Formation of lactic acid and nitrosamine	Smith et al. (1992)
	*Rahnella aquatilis*	Undesirable formation of diacetyl & dimethyl sulphide (DMS)	Van Vuuren et al. (1980)
	*Citrobacter freundii*	Notable off-flavours and aromas. Production of diacetyl, DMS, acetoin, acetaldehyde, lactic acid and 2,3-butandiol	Van Vuuren et al. (1980)
	*Klebsiella terrigena*	High concentrations of DMS	Van Vuuren et al. (1980)
	*Klebsiella oxytoca*		
Fermentation	*Ped. inopinatus*	Longer fermentation times, production of diacetyl	Priest and Campbell (2003)
	*Selenomonas lacticifex*	Relatively strong beer spoilage potential	Schleifer et al. (1990)
	*Zym. paucivorans*	Poorly characterised as a beer spoilage microorganism. Reported to not grow in beer at pH 4.6 and at ethanol concentrations <5% (w/v)	Schleifer et al. (1990); Suzuki (2020)
	*Zym. raffinosivorans*	Spoilage activity similar to *Pectinatus* spp., turbidity, production of H_2_S and “rotten-egg” smell	Jespersen and Jakobsen (1996)
	*Rahnella aquatilis*	Notable off flavours and aromas from production of diacetyl, DMS, acetoin, acetaldehyde, lactic acid and 2,3-butandiol	Van Vuuren et al. (1980)
	*Obesumbacterium proteus*	Inhibit fermentation, increased beer pH, production of acetoin, lactic acid, propanol, DMS, isobutanol and 2,3-budandiaol Parsnip-like off-flavour and aroma	Priest et al. (1974)
	Non-Saccharomyces*	Slow or stuck fermentations, superattenuation. Final beer: turbid, off-flavours & aromas	Lawrence (1988)
	*Saccharomyces* spp.**		
Ageing, Filtration, Pasteurisation & packaging	*Meg. cerevisiae*	Risk to no and low alcohol beers. Unpleasant odours. Formation of acetoin, hydrogen sulphide, butyric, acetic, caproic, isovaleric and valeric acids.	Back (2005)
	*Pect. cerevisiiphilus*	Haziness reported, produce vast amounts of acetoin, hydrogen sulphide, acetic and propionic acids. Sour taste, “rotten-egg” smell	Paradh et al. (2011)
	*Pect. frisingensis*		
	*Lactobacillus* spp.	Hazing or ropiness, sourness, undesirable flavours, and aromas	Suzuki (2015)
	*Pediococcus* spp.		
Contaminants of final product	*P. damnosus*	Off-flavours: production of diacetyl and lactic acid. Turbidity, acidity, gas production, ropiness	Sakamoto and Konings (2003)
	*P. inopinatus*	Minor beer spoilage potential	Iijima et al. (2007)
	*L. brevis*	Turbidity, acidity, superattenuation, gas production and off flavours	Suzuki (2011a)
	*L. casei*	Diacetyl off-flavour in final beer	Suzuki (2015)
	*L. coryneformis*		
	*L. plantarum*		
	*Meg. cerevisiae*	Production of butyric acid, H_2_S, and small amounts of C-5 and C-6 fatty acids. Turbidity and unpleasant off-flavours	Juvonen (2015); Paradh et al. (2011)
	*Mic. kristinae*	Uncommon aroma of fruitiness in beer	Back (1981)
	*Z. mobilis*	Production of large quantities of acetaldehyde and hydrogen sulphide. ‘Fruity’ and ‘sulphidic’ aroma characteristics	Anderson and Howard (1974); Dadds et al. (1971)
Dispense	*A. aceti*	Haziness, acidification (lactic and acetic acid strains), production of diacetyl, unpleasant phenolic, buttery, rotten egg and atypical fruity aromas	Quain (2015)
	*A. pasteurianus*		
	*G. oxydans*		
	Lactic acid bacteria		
	Wild yeasts		

^a^The information is summarised from the literature cited in the table. ^b^L., Lactobacillus, Meg., Megasphaera, P., Pediococcus, Pect, Pectinatus, Mic., Micrococcus, Z, Zymomonas, Zym, Zymophilus. *Non-Saccharomyces wild yeasts include organisms of the genera Brettanomyces, Candida, Debaryomyces, Dekkera, Filobasidium, Hanseniaspora, Kluyveromyces, Pichia, Torulaspora, Zygosaccharomyces. **Saccharomyces wild yeasts primarily include three species, S. cerevisiae, S. bayanus and S. pastorianus.

### Beer spoilage organisms

#### Gram-positive spoilage bacteria

The most significant Gram-positive bacteria associated with the brewing process are lactic acid bacteria (LAB), and these organisms are arguably the most frequently encountered contaminants within the brewery. The LAB designation reflects a range of related organisms that are typically facultative anaerobes, catalase-negative, non-spore-forming, non-motile, acid-tolerant, and can be either rod or cocci in shape. Key examples include *Lactobacillus* and *Pediococcus* species; those associated with beer are strictly fermentative and share the ability to produce haze and diacetyl, as well as acids as a product of fermentation (Pfeiler and Klaenhammer, 2007). In the case of homofermentative LAB, lactic acid is the primary metabolite, while heterofermentative organisms are also able to yield acetic acid, ethanol and carbon dioxide. Consequently, although LAB can be exploited in traditional spontaneous-fermentation styles such as lambic beers (providing typical ‘sour’ flavours via production of acids), most LAB found cause negative effects, resulting in an undesirable or substandard product (Bergsveinson and Ziola, 2017). *L. brevis* and *P. damnosus* are particularly well-known brewery spoilage organisms, although others such as *L. lindneri* and *L. casei* are also found (Table 2). Lactic acid bacteria found across the brewing chain tend to comprise isolates that have developed hop resistance strategies, an unusual characteristic not typically found in gram positive microorganisms, a primary reason why they are among the most common beer spoilage organisms.

#### Hop resistance in LAB

Beer contains bitter compounds (*cis-* and *trans-*isohumulones), derived from the hop plant *Humulus lupulus* L. Although bitterness is an important sensorial property of beer, hop compounds (predominately *iso-α-*acids) also play an additional role in brewing as they exert antimicrobial activity, acting as biopreservatives. Hop bitter acids act as protonophores and inhibit the growth of Gram positive bacteria and hop sensitive LAB strains by dissipating the transmembrane pH gradient (Simpson, 1993a,b), while simultaneously binding important divalent cations that can act as enzyme cofactors, such as manganese, inside the cell. In LAB, a disrupted transmembrane pH gradient inhibits proton motive force (PMF), the mechanism by which LAB strains generate energy (ATP) and transport nutrients (Kashket, 1987). Beer-spoilage LAB have evolved to display resistance to antimicrobial hop bitter acids, a trait that is rarely seen in other Gram-positive species prevalent in foods, such as *Bacillus* and *Staphylococcus*. Consequently, it should be stressed that hop resistance in beer-spoiling LAB is not a species trait *per se*, but rather an isolate-specific ability. To support this, it is known that strains of *L. brevis*, a major culprit of beer spoilage (Back, 2005), shows variability in beer spoilage potential. Specifically, *L. brevis* isolates from sources other than brewing environments generally exhibit no or weak beer spoilage ability (Suzuki, 2009, 2011b). Hop resistance is therefore recognised as a distinguishing character responsible for intraspecies differences in LAB beer spoilage ability (Simpson and Fernandez, 1992; Fernandez and Simpson, 1993; Suzuki et al., 2005c). Until relatively recently, it was believed that hop resistance was a stable character coded by chromosomal DNA (Simpson and Fernandez, 1992; Fernandez and Simpson, 1993; Simpson, 1993a). However, studies have since shown that hop resistance of lactobacilli decreases upon serial subculturing in the absence of hop compounds; in contrast hop resistance increased 8- to 20-fold in hop-resistant strains of lactobacilli upon serial subculturing in media supplemented with increasing concentrations of hop bitter acids (Shimwell, 1936; Richards and Macrae, 1964; Suzuki et al., 2006). These studies emphasised that hop-resistance observed with LAB strains is unstable in nature (Suzuki et al., 2004a,b, 2005a,c). Furthermore, hop resistance genes and the genetic markers for beer spoilage LAB strains have since been shown to occur on mobile DNA units; both plasmids and putative transposons (Suzuki, 2009; Geissler et al., 2017). The horizontal transfer of hop resistance genes is believed to be one of the influencing factors behind the emergence of newly recognised beer spoilage LAB species in beer (Suzuki et al., 2006; Suzuki, 2009, 2020; Umegatani et al., 2022), and offers a broader threat to the industry in the future.

The genetic elements currently associated with hop resistance are *horA, horC, hitA* and ORF5. The *horA* and *hitA* genes code for primary- and secondary-type multidrug transporters, respectively (Sami et al., 1997; Suzuki et al., 2005b). The *horA* gene has homology to ATP-binding cassette-type multidrug resistance genes, whereas it has been suggested that *hitA* is a hop-inducible divalent cation transporter (Hayashi et al., 2001). *horC and* ORF5 genes code for proteins of unknown function with no homology to known proteins. ORF5 is believed to be a putative PMF-dependent multidrug transporter (Suzuki et al., 2004a,c); however, this genetic marker has not been extensively evaluated for the differentiation of beer spoilage ability of LAB species (Suzuki et al., 2005a). Due to the discovery that *horB*, the regulator of *horC*, encodes a regulator with homology to AcrR regulators (involved in controlling the transcription of genes that encode multidrug transporters; Suzuki et al., 2005a), it is believed that *horC* encodes a multidrug transporter (Iijima et al., 2006). Most, if not the majority of, the newly emerging beer spoilage LAB species possess one of these genetic markers. The combined application of the trans-species genetic markers *horA* and *horC* is considered a useful technique for detecting these uncharacterised beer-spoilage LAB species (Deng et al., 2014). However, it should be noted that beer spoilage LAB strains that do not carry these genetic markers have also been reported (Haakensen et al., 2008; Bergsveinson and Ziola, 2017). A comprehensive overview of the antimicrobial activity of hops and specifically the interaction between hops and LAB has been published by Suzuki (2011a).

#### Aerobic gram-negative spoilage bacteria

Gram-negative bacteria commonly associated with beer include a number of aerobic and facultatively anaerobic organisms, including acetic acid bacteria (AAB), *Zymomonas* species and certain *Enterobacteriaceae*. These aerobic bacteria are, by definition, not typically an issue for packaged beer in which oxygen has been effectively excluded. They are, however, commonly found post-packaging, associated with opened kegs or draft beer dispensing lines where correct hygiene and practices to limit oxygen entry are not respected (Ziola and Bergsveinson, 2017).

*Acetic Acid Bacteria* are problematic due to their ability to produce and tolerate acidity, and to the assortment of substrates that they can metabolise (including glucose, ethanol, lactate, and glycerol). However, out of 15 confirmed genera, only two: *Acetobacter* and *Gluconobacter,* are reported to be associated with beer spoilage (Paradh, 2015). Within these genera, *G. oxydans, A. aceti* and *A. pasteurianus*, are well-known brewery spoilage organisms (Table 2). *Gluconobacter* and *Acetobacter* convert ethanol to acetic acid, which gives an unpleasant vinegary off-flavour to beer. Furthermore, growth of *Gluconobacter* in beer can result in the production of a pellicle on the surface, and eventual haziness. Related to this, some strains of *Gluconobacter* can also cause ropiness in beer due to the production of dextrans and levans, which can also increase the viscosity of beer (Hornsey, 1999). The AAB found in breweries are generally resistant to hop compounds, can survive in high concentrations of ethanol (>10% v/v) and are acidophilic. Due to these characteristics, AAB have the potential to occur throughout the brewing chain, specifically at locations where oxygen is present. Furthermore, AAB are often associated with other beer spoilage organisms in the form of biofilms that are able to accumulate in niches and corners in brewery filling equipment and dispensing lines. Nowadays, aerobic AABs pose a limited threat within most large breweries, due to improved technology leading to a radical decrease in the O_2_ content in final beer, and the routine implementation of successful cleaning and sanitation practices. The decline in spoilage incidents has resulted in AABs being frequently regarded as non-critical in the brewery. However, risks for smaller companies, typically those producing ale type products may remain, while their presence in general can be an indicator of inadequate sanitation and hygiene (Sakamoto and Konings, 2003). AAB do however remain problematic at the point of sale and can frequently be isolated from dispense systems in pubs and public houses, due to the presence of oxygen and elevated temperatures encountered (Storgards, 2000). For similar reasons, regular occurrences of beer spoilage in draught beer kegs have been described, and AAB are still common in cask-conditioned and barrel-aged beers; 46% of the microflora isolated from cask ale samples were identified as acetic acid bacteria in a recent study by Jevons and Quain (2022).

*Enterobacteria* are a substantial family of Gram-negative facultatively anaerobic bacteria, that comprise both pathogenic and non-pathogenic genera. Being facultative anaerobes, members of this family are able to grow in the presence or absence of air, however they are also typically inhibited by ethanol and low pH (Priest, 2006). Because of this, they are almost exclusively found as wort spoilers, often arriving via contaminated water (Table 2). The *Enterobacteriaceae* isolated from brewery environments are all non-pathogenic and include *Citrobacter, Hafnia, Klebsiella* and *Obesumbacterium* species. These microorganisms are able to proliferate in nutrient-rich wort during the early stages of fermentation with a pH of approximately 5–6. Their presence can cause undesirable off-flavours due to the production of DMS, providing a parsnip-like sulphury flavour to beer (Priest, 2006), as well as 2,3- butanediol, acetate, formate, and low levels of fusel alcohols (Priest et al., 1974; Priest and Hough, 1974; Van Vuuren et al., 1980). Subsequently, the final product can have sweet ‘fruity’ or vegetable-like (celery or cooked cabbage) off-flavours (Ziola and Bergsveinson, 2017).

*Zymomonas* species are common spoilage microorganisms encountered in a variety of traditional alcoholic beverages globally (Coton et al., 2006). Bacteria of this genus have a unique mode of catabolism, carrying out highly efficient ethanolic fermentation, and rendering it a biotechnologically relevant microorganism for industrial production of fuel ethanol (Gírio et al., 2010; Chandel et al., 2011). As might be expected the bacterium is ethanol tolerant (≤ 10% v/v), and grows optimally at a pH greater than 3.4 and prefers temperatures of 25–30°C (Van Vuuren and Priest, 2003). At present *Zymomonas* has only one species (*Z. mobilis*; Table 2), originally isolated from beer (Shimwell, 1937). However, *Z. mobilis* has three validated subspecies, of which only *Z. mobilis* subsp. *mobilis* is reported to be a beer spoiler (Van Vuuren and Priest, 2003). *Zymomonas* spoilage is a frequent occurrence in the cider industry, causing off-flavours described as rotten banana, grassy, rotten lemon, or raspberry. *Z. mobilis*-contaminated beer has a similar unpleasant aroma, due to the production of acetaldehyde and hydrogen sulphide. The source of contamination by this species is still largely unknown, however, soil is considered to be a possible reservoir (Paradh, 2015). Due to the relatively strict carbohydrate requirements (glucose, fructose, sucrose and raffinose), the presence of this microorganism is restricted to ale breweries where priming sugars are regularly employed. *Zymomonas* is not associated with breweries producing lager type beers (Dadds and Martin, 1973), supported by the absence of reports in more recent times.

#### Anaerobic gram-negative spoilage bacteria

In the early 1990s, improvements to filling technology deigned to enhance flavour stability and shelf-life by limiting oxygen content in packaged beer led to a sudden increase in reports of spoilage caused by strictly anaerobic bacteria of the class *Clostridia*. This group of microorganisms currently comprise nine species that are distributed between the genera *Megasphaera, Pectinatus, Selenomonas* and *Propionispira* (Juvonen, 2015). These microbes pose a severe threat since they are predominately isolated from finished and packaged beer (Back, 1994), and especially products that are unpasteurised with an elevated pH (Ziola and Bergsveinson, 2017). Although, the natural environment and source of these spoilage organisms is not entirely understood, their presence can most likely be attributed to the development of anaerobic regions within biofilms, arising through the symbiotic relationships of the microorganisms within them. Irrespective, spoilage is characterised by development of turbidity, sour tastes, and a range of obnoxious odours that render the product inconsumable (Suzuki, 2011a), causing serious economic losses and, if recalled from the market, detrimental reputational damage to a brand. Beers tainted by *Megasphaera* form only modest hazes in beer and virtually indetectable sediments. However, unpleasant aroma compounds including hydrogen sulphide, butyric- and caproic acid (commonly: ‘baby sick’ and ‘waxy/goaty’, respectively) are formed that necessitate destruction of the product (Back, 2005). In comparison, beers contaminated by *Pectinatus* present heavy sediments, hazes, and yield extremely unpleasant taste and odours due to the production of hydrogen sulphide. As spoilage occurs in the latter stages of processing, the economic losses and brand damages caused can be significant; spoilage incidents by *Pectinatus* and *Megasphaera* are arguably the most feared within the industry. Different factors affect the beer spoilage ability of these strict anaerobes and it has been noted that beers with a lower alcohol content are more susceptible to contamination; *Pectinatus* and *Megasphaera* are not able to grow in beers with an ethanol content exceeding 5.2 and 3.5% (w/v), respectively (Haikara and Helander, 2006). However, as alluded to above, the most essential determinant for permissive growth of *Pectinatus* and *Megasphaera* is the low oxygen content of beer, although initial contamination is usually due to ineffective sterilisation and poor hygiene in packaging lines accompanies by ineffective pasteurisation.

The genus *Propionispira* has two brewery-associated species that are both obligately anaerobic: *P. paucivorans* and *P. raffinosivorans* (Schleifer et al., 1990). These species have been reallocated to this group (*previously Zymophilus*) based on 16S rRNA sequence homology (Ueki et al., 2014) and, along with *Selenomonas lacticifex*, are predominately isolated from pitching yeast. Although *Propionispora* and *Selenomas* isolates are morphologically similar and phylogenetically related to *Pectinatus* (Schleifer et al., 1990), the beer spoilage ability of these organisms is relatively obscure. From laboratory inoculation tests, *S. lacticifex* has been reported to grow in beer with a pH value of 4.3–4.6, indicating that this species should be classified as an obligate or potential beer spoiler (Juvonen et al., 2008; Suzuki, 2011a). In comparison, *P. raffinosivorans* and *P. paucivorans* are only capable of growth in beer with pH 5.0 and 6.0, respectively (Suzuki, 2011a). This suggests that *P. raffinosivorans* may be a direct potential beer spoiler, whereas *P. paucivorans* is more likely to act as an indicator microorganism for poor hygiene in the brewery, rather than spoil beer directly.

#### Fungal contaminants within the brewing chain

As described above, brewing raw materials including barley, malt, hops, and adjuncts have their own microbiota. The barley-associated microbiota is believed to be largely made up of fungi and moulds (Vaughan et al., 2005) and bio-diversity is determined by the field conditions under which the crop was grown, together with the post-harvest handlings of the grain (Flannigan, 1996b). Contamination of barley at any stage may adversely impact the quality of the malt, wort and beer (Lawrence, 1988). Species of *Alternaria, Cladosporium, Epicoccum* and *Fusarium*, are primary examples of field fungi (Flannigan, 1996b). *Fusarium* spp. are particularly important as they account for *Fusarium* head blight (or scab), which can influence both yield and the functional parameters of grain associated with malting and therefore brewing quality (Nielsen et al., 2014). *Fusarium* spp. such as *F. graminearum* and *F. culmorum* can also cause food safety issues, as they are able to produce toxic secondary metabolites known as mycotoxins (Vaughan et al., 2005). Mycotoxins, such as deoxynivalenol (DON) and zearalenone (ZEA), can be found in many cereal-based products (Chelkowski, 1989) and cause a range of harmful health effects (such as vomiting) that pose a health threat to both humans and livestock (WHO, 2018). Schwarz et al. (1995) and Scott et al. (1993) demonstrated that *Fusarium* toxins can be formed during the malting process and subsequently transmitted into the finished beer. Aside from negative health impacts, certain *Fusarium* species are known to also form compounds that act as active gushing inducers. Gushing is a complex phenomenon whereby spontaneous excessive foaming of beer occurs when opening bottles, cans or kegs (Laitila et al., 2002). This occurrence can, to some extent, be explained by the release of specific factors (hydrophobins that serve to nucleate and stabilise bubbles) from fungi on barley in the field, during storage or during the malting process (Amaha and Kitabatake, 1981; Munar and Sebree, 1997).

Within the brewery, beer-spoiling yeasts represent a greater and more persistent challenge. These organisms represent a highly varied group that have the potential to cause a range of detrimental effects to beer, either by impacting process stages or by altering the character of the beer directly. In general, beer-spoiling yeasts show tolerance to ethanol and resistance to low pH. Defects include the formation of phenolic compounds, acidity, fatty acid compounds and high-ester off-flavours, as well as hazes and turbidity (Lawrence, 1988). A further impact is related to the performance of the culture yeast; most beer-spoiling yeast strains do not perform or sediment in the same way as production strains, often generating a different portfolio of flavour compounds and presenting a weaker flocculation potential. Furthermore, many spoilage yeasts do not interact with finings as they lack a strong negative charge; as a result beers can appear cloudy in nature and may also exhibit off-flavours due to cell lysis (Powell and Kerruish, 2017). Beer-spoiling yeasts can be divided into non-fermentative (aerobic) yeasts, predominately associated with raw materials and process steps where oxygen exclusion is hard to implement; and fermentative (anaerobic) yeasts, that are able to compete with production strains during fermentation (Table 3). Characteristics of aerobic and anaerobic beer- spoiling yeasts. Alternatively they can be divided into *Saccharomyces* and non-*Saccharomyces* types, since these 2 groups have broadly distinct effects (Back, 1987, van der Aa Kühle and Jespersen, 1998). The *Saccharomyces* spoilage yeasts are often deemed to be the most ‘dangerous’ due to their difficulty to detect, and their ability to directly compete with the culture strain. Among the non-*Saccharomyces* species, the following genera predominate: *Brettanomyces*, *Candida*, *Debaryomyces*, *Hanseniaspora* (*Kloeckera*), *Kluyveromyces*, *Pichia*, *Torulaspora*, and *Zygosaccharomyces* (Table 3). Unlike some of the spoilage bacteria mentioned previously, beer-spoiling yeasts are not normally exclusive to industrial locations but are associated with casks and raw materials such as hops, priming sugars and adjunct syrups. These materials act as a source of entry into the brewery, leading to contamination across various stages of the brewing process. Therefore, brewing equipment, surfaces, water supplies, and pitching yeast can be deemed potential sources of contamination.

**Table 3:** Characteristics of aerobic and anaerobic beer- spoiling yeasts.

Metabolism	Common species	Descriptions	Beer spoilage potential
Aerobic	*B. anomalus* (*D. anomola*) *B. bruxellensis* (*D. bruxellensis*) *B. lambicus*	Elongated cell structure, can form short chains Facultative anaerobes Not able to ferment sucrose Limited fermentation with maltose Desirable for production of certain beer types	Produces acetic acid, volatile fatty acids, 4-EP (barnyard, medicinal character) and 4-EG (spice, cloves, smoky) Can form pellicles Off-flavours, especially for bottle-conditioned beers Ethanol tolerant and resistant to low pH Detected in unpasteurised draught beer
	*C. boidinii* *C. stellata* *C. tropicalis* *C. vini*	Spherical-shaped cells Ferments glucose, occasionally maltose Limited growth under anaerobic conditions	Fermentation, turbidity, and off-flavours Can oxidise ethanol to produce acetic acid Can form pellicles Spoilage mainly during aerobic stages of production
	*Db. hansenii*	Small spherical cells Weak or no fermentation Tolerant to cold, salt and changes in osmolality	Turbidity and yeasty off-flavours Can form pellicles or deposit Spoilage mainly during aerobic stages of production
	*L. saturnus*	Spherical-shaped cells Limited fermentation Explored for production of low alcohol, fruity wines	Produces strong estery flavours Often associated with killer activity
	*P. anomola* *P. fermentans* *P. membranifaciens*	Ovoid/ellipsoidal, or rod-shaped cells Favour aerobic conditions Fermentation weak or absent, can only ferment glucose Cell function inhibited by alcohol and low pH	Turbidity and yeasty-off flavours May lead to an increase in ester production Can form pellicles or deposit Associated with draft beer, raw materials, or the early stages of fermentation
	*R. glutinis* *R. mucilaginosa*	Typically, ovoid/ellipsoidal May produce pseudohyphae Fermentation absent Can survive in cold conditions and aerobic environments	Able to assimilate sugars, resulting in decreased efficiency of fermentation Able to persist in pitching yeast but usually does not result in beer spoilage Route of entry may be via malt
Anaerobic	*H. uvarum* (*K. apiculata*) *H. valbyensis* *H. vineae*	Apiculate yeast Fermentative Preference for anaerobic conditions	Fermentation, turbidity and off-flavours More commonly associated with grapes, but may be present due to cross contamination
	*K. marxianus*	Assorted cell morphologies, typically ovoid/ellipsoidal Fermentative Some strains contain killer plasmids that can negatively impact pitching yeast culture and fermentation	Fermentation, turbidity and off-flavours
	*S. bayanus* *S. cerevisiae* *S. pastorianus* *S. unisporus*	Spherical-shaped cells (*S. bayanus* may have elongated cells) Includes non-production strains and variants Fermentative	Fermentation, turbidity, and off-flavours Certain strains have varying flocculation (POF+) that can interact with brewing yeast Certain strains can produce phenolic off-flavour compounds Diastatic yeasts can result in low attenuation and decreased mouthfeel
	*Sch. pombe*	Rod-shaped cells Fermentative Utilised in traditional African beers	Fermentation, turbidity, and off-flavours
	*T. delbrueckii*	Spherical/ellipsoidal cells Fermentative (certain species ae obligate fermenters) Grows poorly under anaerobic conditions Well adapted to tolerate osmotically challenging environments, associated with contamination of raw materials such as priming sugars	Fermentation, turbidity, and off-flavours Associated with pitching yeast Able to contaminate unpasteurised beer
	*Z. bailii* *Z. bisporus* *Z. rouxii*	Oval-shaped cells Fermentative High sugar tolerance and survive in extreme sugar concentrations, potentially contaminating syrups, and adjuncts	Fermentation, turbidity, and off-flavours Produce higher alcohols and yeasty off-flavours

^b^B., Brettanomyces; D., Dekkera; C., Candida; Db., Debaryomyces; L., Lindnera; P., Pichia; R., Rhodotorula; H., Hanseniaspora; K., Kluyveromyces; S., Saccharomyces; Sch., Schizosaccharomyces; T., Torulaspora; Z., Zygosaccharomyces.

## Microbiological opportunities and challenges for no- and low-alcohol beers

### Microorganisms for the production of no- and low-alcohol beer

The rising demand for alcohol-free products has aided the development of strategies and technologies suitable for NoLo beer production at large scale. Approaches to creating NoLo products can be broadly divided into physical and biological processes: physical methods involve the removal of ethanol from regular alcoholic beer (de-alcoholisation), whereas biological processes are dependent on restricting ethanol formation (Brányik et al., 2012). Although effective, both approaches can create organoleptic imperfections by altering flavour profiles (Blanco et al., 2016). This occurs either due to the loss of important flavour active components (along with the ethanol fraction) during de-alcoholisation, or due to poor or incomplete flavour development under limited fermentation, especially when using standard brewing yeast strains. Due to this, the exploration of non-conventional (i.e., non-*Saccharomyces*) yeasts with a reduced ability to ferment wort sugars has attracted growing research interest (Bellut and Arendt, 2019). Given that maltose and maltotriose typically constitute around 80% of the total carbohydrate content in standard wort types, yeasts that cannot assimilate or utilise these sugars (i.e., maltose- and maltotriose-negative yeasts) can be used to yield beer with a low alcohol content. Residual sugars in the final product are not necessarily detrimental, since this can contribute towards body and sweetness, which can be considered positive in some beer styles. The concept of using ‘special’ yeasts was first patented by Glaubitz and Haehn (1933) using a strain of *Saccharomycodes ludwigii*, but has gained traction in recent years with further patents centred around the use of both *S. ludwigii* (Kunz and Methner, 2009) and *Pichia kluyveri* (Saerens and Swiegers, 2014). More recent investigations on the use of non-*Saccharomyces* yeasts for production of NoLo beers have included the yeasts *Candida shehatae* (Li et al., 2011)*; Cyberlindnera* spp. including *C. fabianii* (van Rijswijck et al., 2017) and *C. mrakii* (formerly *Williopsis saturnus* var. *mrakii*; Liu and Quek, 2016); *Hanseniaspora* spp. including *H. valbyensis* and *H*. *vineae* (Bellut et al., 2018); *Pichia kluyveri* (Saerens and Swiegers, 2014; *Pichia kudriavzevii* (van Rijswijck et al., 2017); *Torulaspora delbruickii* (Canonico et al., 2016; Michel et al., 2016; and *Zygosaccharomyces* spp., including *Z. rouxii* (Sohrabvandi et al., 2009; De Francesco et al., 2015), *Z. bailii and Z. kombuchaensis* (Bellut et al., 2018). In each instance, fermentation variables must be manipulated to ensure process efficiency while also encouraging poor conversion of sugars into ethanol; a challenging juxtaposition since these are conflicting goals under normal circumstances. Precise strategies are inevitably species (and strain) dependent, but typically involve manipulating oxygenation during fermentation, elevating or reducing fermentation temperature, reducing initial wort gravity and adjusting pitching (inoculation) rates (Mortazavian et al., 2014). It should be noted that although the principle aims of producing NoLo beer can be achieved using a growing array of yeast types, most products display flavour imbalance when compared to ‘traditional’ beers. Sensory descriptive analysis of no-alcohol beers produced with non-*Saccharomyces* strains often highlight ‘worty’ notes, as well as an imbalance of esters and higher alcohols. However, unusual sweet and fruity notes including apricot, lychee, pear and citrus have also been reported; although these flavours are not typically associated with beer, consumer studies have shown a preference for no-alcohol beers which do exhibit lightly fruity aroma (Schmelzle et al., 2013). The application of non-*Saccharomyces* yeasts for NoLo brewing may therefore prove to be an opportunity to create ‘stand-alone’ products with novel flavours, as well as attempting to re-create beverages that match regular beer brands.

### Microbial contamination of no- and low- alcohol beer

Standard beer represents a microbiologically stable environment, due to the presence of hop bitter acids, low pH, reduced oxygen, and elevated concentrations of carbon dioxide. Furthermore, a lack of nutrients and the presence of ethanol are not commensurate with microbial proliferation. However, NoLo products often contain higher residual extracts, exhibit elevated pH levels, and tend to be weakly hopped, reducing the effectiveness of the intrinsic hurdles that would normally protect against microbial spoilage. Consequently, there is a threat to the industry that microorganisms previously believed to be irrelevant to the brewing environment may be able to survive and proliferate in final pack, which could give rise to a surge in microbiological beer-spoilage incidents (Kurniawan et al., 2021).

In order to determine the sensitivity of NoLo beers to spoilage, Riedl et al. (2013) performed a ‘risk assessment’ based on seven distinct microorganisms including spoilage yeast and bacteria (detailed in Table 4). Each organism was inoculated into bottles of no-alcohol beer (ale and lager), low-alcohol beer (ale and lager) and standard beer (ale and lager), all of which were incubated at 28°C using an adapted forcing test. Visual assessments were performed periodically for gas formation, haze, biofilm formation and agglomeration, and finally cell concentration was determined after 28 days. The results obtained allowed each microorganism to be assigned a risk category based on growth, ranging from 0 (no growth) to 3 (strong growth) and the sum of these values were used to indicate overall risk (Table 4). According to this study, the two no-alcohol beers (NoB1 and NoB2) were classified as ‘very sensitive’, as all microorganisms showed positive growth (except *Lactobacillus brevis* in NoB1; subsequently shown to be sensitive to hops). Similarly, the microbiological risk of low-alcohol beers was classified as being ‘high’, while the regular beer was classified as ‘microbiologically stable’, even though *Saccharomyces cerevisiae* var. *diastaticus* yeasts and *Dekkera anomalus* were able to proliferate. Beyond this study, it is possible to make predictions on the spoilage potential of specific organisms based on current understanding of environmental preferences. For example, it is known that ethanol-sensitive species such as *Gluconobacter* spp. are able to grow well in sugar-rich environments and are associated with spoilage of soft drinks at low pH (Raspor and Goranovič, 2008). These strains are therefore likely to pose a contamination risk to NoLo beers, especially those characterised by elevated residual extracts and low pH. Furthermore, studies have shown that *Gluconobacter* spp., are resistant to preservatives such as sorbic acid, benzoic acid and dimethyldicarbonate (Raspor and Goranovič, 2008), although their dependence on free oxygen offers a means of control. Similarly, the ability of *Z. mobilis* to metabolise glucose, fructose (and sometimes sucrose) to equimolar quantities of ethanol and CO_2_ could be detrimental in NoLo beers that are produced via limited or arrested fermentations, due to the high fermentable sugar content of these products. Based on product type, it may be possible to implement potential strategies to prevent spoilage such as pH reduction, especially since *Z. mobilis* shows poor growth potential at pH values below 3.5 (Coton et al., 2005). On a similar theme, *Enterobacteriaceae* species are unable to survive at high ethanol concentrations but often show excellent growth in its absence. For example, *O. proteus*, *C. freundii* and *R. aquatilis* are not commonly found in standard beer types (Paradh, 2015), but the ethanol-free, nutrient dense environment associated with the production of no-alcohol beers could promote the growth of these microorganisms. Importantly, contamination by organisms such as *O. proteus* may pose a risk to consumers as these microorganisms are able to produce *N*-nitrosamines; carcinogenic compounds that can cause health risks if consumed in excess (Smith, 1994). Typically concentrations of *N-*nitrosamines are monitored in breweries (Maugueret and Walker, 2002), but it is extremely rare that they exceed the specified limits. However, NoLo products may warrant greater focus to ensure compliance with regulations and to minimise risk associated with this hazard.

**Table 4:** Risk assessment comparing microbiological sensitivity of NoLo beers (Riedl et al., 2013).

	NoB		LoB		Beer	
Beverage	NoB1 BF	NoB2 TF	LoB1 BF	LoB2 TF	B1 BF	Std
Microorganisms						
*L. brevis*	0	3	0	3	3	3
*Pectinatus portalensis*	3	3	0	0	0	0
*S. cerevisiae*	3	3	3	1	1	0
*S. pastorianus ssp. carlsbergensis*	3	2	3	2	2	0
*S. cerevisiae var. diastaticus*	3	3	3	3	3	3
*Dekkera anomola*	3	3	3	3	3	3
*Wickerhamomyces anomalus*	3	3	2	3	2	0

Individual values represent ‘risk category’, where 0 indicates low risk (no growth) and 3 indicates high risk (strong growth). Where: NoB, non-alcoholic beer; LoB, low-alcohol beer; TF, top-fermented; BF, bottom-fermented; L., Lactobacillus; S., Saccharomyces. Note that the standard full alcohol beer B1 BF is described as a dark beer with elevated residual extract, while the product denoted as ‘Std’ refers to a standard Helles lager style product.

Finally, there are some bacterial species not normally associated with beer that may become problematic in the future. One example is the identification of *Clostridium acetobuylicum*, found (rarely) as a contaminant of NoLo beer (Back et al., 1992; Suzuki, 2011a), most likely due to their capacity to grow at relatively low pH values of less than 4.2. More importantly, it would be remiss to ignore risks associated with the growth and survival of pathogenic microorganisms. It is widely accepted that pathogens are not able to survive in beer, and various studies have demonstrated that their survival is generally poor or absent completely (Felsenfeld, 1965; Sheth et al., 1988; Menz et al., 2009). Furthermore, focused studies examining the growth and survival of pathogenic *Enterobacteriaceae* (including species such as *Escherichia*, *Klebsiella*, *Salmonella*, *Serratia* and *Shigella*) have shown that these organisms are specifically inhibited due to the antimicrobial hurdles associated with beer (Menz et al., 2009, 2010). Despite this it is recognised that pathogen survival and/or development is inevitably improved when antimicrobial hurdles are lessened. For example L'Anthoën and Ingledew (1996) confirmed that some food-related pathogens, including, *E. coli* 0157:H7, *Salmonella typhimurium*, *Pseudomonas aeruginosa, Klebsiella pneumoniae*, and *Yersinia enterocolitica* show an ability to multiply in un-pasteurised alcohol free (0.5% ABV) beverages. This early study is supported by more recent data presented by Menz et al. (2011), which indicated that both *E. coli* O157:H7 and *Salmonella typhimurium* were capable of active growth in no-alcohol beers, but that by lowering the pH of the beer to below 4, growth was prevented. These results are supported by a more recent study by Çobo et al. (2023), who found that *E. coli* O157:H7 and *Salmonella enterica* survived in beer for more than 2 months when stored at 4 and 14°C, irrespective of pH (4.20, 4.60 and 4.80) and ABV (0.5 and 3.2%). Furthermore, the research showed that *E. coli* O157:H7 and *S. enterica* were able to grow in no-alcohol beers (approximately 2.00 log) at 14°C in all pHs investigated, but no growth was detected at 4°C. Interestingly analysis of *Listeria monocytogenes* indicated that this organism was more susceptible to these conditions and was not able to proliferate. This analysis indicates that efficient storage and handling of products may become increasingly important in reducing the risk of contamination by microorganisms. Similarly, it is also important that general plant hygiene, cleaning in place (CiP) procedures, production checks, and stringent filler hygiene assessments are rigorously applied to NoLo production streams. For example, beer residues should be reduced and removed during production runs, and additional monitoring systems should be implemented to detect biofilm-forming bacteria (e.g., acetic acid bacteria) and yeast (e.g., *Wickerhamomyces anomalus* and *Saccharomyces* strains) and to analyse final products as part of routine quality control analysis. It may also be prudent to ensure that the microbial loading of NoLo products is controlled post-packaging (i.e., using tunnel pasteurisation) rather than implementing strategies that reduce microbial loading prior to filling containers (i.e., sterile filtration and flash pasteurisation; Suzuki, 2020).

Studies assessing the susceptibility of NoLo beers to spoilage have been limited to those identifying effective pasteurisation strategies and the unsurprising observation that ethanol augments heat in killing microorganisms. Experiments using spoilage yeasts (Quain, 2021), yeast spores (Rachon et al., 2021), and bacteria (Adams et al., 1989; L'Anthoën and Ingledew, 1996) have all demonstrated that microbes are more tolerant to heat when alcohol is absent. Thus, NoLo beers in final pack may necessitate enhanced levels of pasteurisation to reach commercial stability than standard beers containing alcohol.

It should also be recognised that in parallel to general commercial growth, there is also increasing consumer demand for draught NoLo beers in the on-trade (Hancock, 2019). This poses an additional level of complexity, since the quality of beer once it has left the brewery is no longer under the control of the brewer but is dictated by handling procedures at the point of dispense (Mallett and Quain, 2019). A series of challenge-test studies performed by Quain (2021) demonstrated that draught beer spoilage was 2–5 times greater in NoLo lager type beer when compared to standard products at 4.5%. Interestingly, spoilage was strongly correlated with the levels of residual fermentable sugars and the availability of micronutrients in the product, rather than the absence/presence of ethanol *per se*. In fact, adding ethanol directly to NoLo beers provided only mild protection against spoilage. These results demonstrate that certain strategies used for NoLo beer production, perhaps where higher residual extracts are present (i.e., arrested/limited fermentation), could render final products more susceptible to spoilage, and may warrant more stringent microbiological control than those that involve ethanol stripping. Furthermore, given the susceptibility of NoLo beers to microbial spoilage and the liability to microorganisms that are typically inhibited by ethanol, NoLo beer products may warrant their own bespoke dispense systems in the on-trade, replacing conventional long line dispense mechanisms. Indeed, Quain (2021) recommended that brewers consider novel, hygienically designed stand-alone dispense system that limit or significantly decreases the risk of microbial contamination, growth, and associated product spoilage.

## Conclusion

Although the positive attributes of microorganisms in brewing vastly outweigh any negative aspects, the increasing popularity of non-traditional beverages poses a challenge to the brewing industry. This is especially true for NoLo beers that have enhanced susceptibility to microbiological spoilage since their composition is fundamentally different to traditional beers. This phenomenon could potentially lead to an increased range of spoilage organisms that may require more extensive sampling procedures and novel identification methods for detection in the future. Fortunately, contamination within the brewing chain very rarely poses a concern with regards to food safety; the presence of microorganisms across the process is an indication of inadequate cleaning and hygiene practices and the greatest concern is with regard to beer quality, including flavour, aroma, and appearance of the final product. However, it is prudent that brewers of all sizes should recognise the potential risk associated with NoLo products and consider the implementation of strategies designed to prevent or mitigate contamination with unwanted organisms.

## Author contributions

GR: Conceptualization, Visualization, Writing – original draft, Writing – review & editing. DK: Writing – review & editing. MC: Writing – review & editing. KS: Writing – review & editing. CP: Conceptualization, Supervision, Writing – review & editing.
